# HIV-1 interaction with an O-glycan-specific bacterial lectin enhances virus infectivity and cell-to-cell viral transfer

**DOI:** 10.21203/rs.3.rs-2596269/v1

**Published:** 2023-02-17

**Authors:** Daniel Heindel, Dania Figueroa Acosta, Marisa Goff, Muzafar Jan, Xiao-Hong Wang, Mariya Petrova, Kun-Wei Chan, Xiang-Peng Kong, Benjamin Chen, Lara Mahal, Barbara Bensing, Catarina Hioe

**Affiliations:** Icahn School of Medicine at Mount Sinai; Icahn School of Medicine at Mount Sinai; Icahn School of Medicine at Mount Sinai; Icahn School of Medicine at Mount Sinai; VA New York Harbor Healthcare System; University of Antwerp; New York University Grossman School of Medicine; New York University Langone Medical Center; Icahn School of Medicine at Mount Sinai; University of Alberta; University of California, San Francisco; Icahn School of Medicine at Mount Sinai

## Abstract

While bacterial dysbiosis has been associated with increased HIV-1 transmission risk, little is known about direct associations between HIV-1 and bacteria. This study evaluated HIV-1 interactions with bacteria through glycan-binding lectins that affect virus infectivity. The Streptococcal Siglec-like lectin SLBR-N, which is part of the fimbriae shrouding the bacteria surface and recognizes α2,3 sialyated O-linked glycans, was noted for the ability to enhance HIV-1 infectivity in the context of cell-free infection and cell-to-cell transfer. SLBR-N was demonstrated to capture HIV-1 virions, bind to O-glycans on HIV-1 Env, and augment CD4 binding to Env. Other SLBRs recognizing distinct O-glycans also enhanced HIV-1 infectivity, albeit to lower extents, whereas N-glycan-binding bacterial lectins FimH and Msl had no effect. Enhancing effects were recapitulated with O-glycan-binding plant lectins. Hence, this study highlights the potential contribution of O-glycans in promoting HIV-1 infection through the exploitation of O-glycan-binding lectins from commensal bacteria at the mucosa.

## Introduction

HIV-1 transmission often occurs through mucosal surfaces during sexual contact, or during the perinatal period, including pregnancy, childbirth and breastfeeding^1^. While bacterial dysbiosis at the mucosa and accompanying inflammation have been associated with an increased risk of HIV-1 transmission, little is known about whether direct interactions between HIV-1 and bacteria that are part of the human mucosal microbiome contribute to HIV-1 transmissibility. There are precedents for intimate connections between viruses and bacteria^2, 3^. Human norovirus, for example, requires a specific histo-blood group antigen (HBGA) expressed by the commensal bacteria *Enterobacter cloacae* to augment infection in B cells; these HBGA glycans bind directly to norovirus particles through the major capsid protein, but their roles in infection are poorly understood^3–5^. Other HBGA-expressing bacteria help protect norovirus from heat stress^6^. Poliovirus binds, via its VP1 capsid, to bacterial lipopolysaccharide (LPS) resulting in increased virus thermostability and resistance to inactivation by chlorine bleach, as well as enhanced binding to the cellular poliovirus receptor^7^. Reovirus also interacts directly with bacterial LPS and peptidoglycan, which augment virus thermostability and attachment to mammalian cells^8^. When mice are depleted of bacterial microbiota by antibiotic treatment before oral exposure to poliovirus or reovirus, they show decreased virus infection and pathology in the intestine^9^. While direct virus-bacteria interactions and their biological consequences have been well studied for these enteric and other viruses, such as murine mammary tumor virus^10, 11^, evidence to show whether HIV-1 can interact with bacteria or bacterial products to impact its infection and transmission is still lacking.

On the virion surface, HIV-1 displays membrane-anchored envelope (Env) glycoproteins with dense glycosylation that occupies most of the Env surface. Each of the three Env monomeric subunits contain both *N*- and *O*-glycans of varying types with heterogeneous compositions. As many as 30 *N*-glycans can be found per Env monomer^12^, serving as a shield masking this sole viral surface antigen from immune recognition^13–15^. Our understanding of *O*-glycans on Env is more limited. Initial reports concerning *O*-glycans on HIV-1 Env were discordant^16, 17^, however a later publication verified the discovery of O-glycans on HIV-1 Env gp120 from different isolates^18^. Specifically, out of the eight mammalian core *O*-glycan structures, core 1 and core 2 structures were identified in the V1 region by mass spectrometry (MS) and the number of predicted *O*-glycosylation sites correlated with the V1 length. An earlier MS analysis of virion-associated gp120 also detected a core 1 *O*-glycan at the highly conserved C5 site located upstream from the furin-cleavage site^19^. The presence of *O*-glycans is not limited to HIV-1, as core 1 and core 2 *O*-glycans have similarly been observed on SIV and HIV-2 gp120s^20^. In a cryo-EM structure of SIV Env in complex with broadly neutralizing antibody (bNAb) PGT145 and *O*-glycan-specific lectin jacalin (AIA), jacalin was found to bind to a V1 *O*-glycan^21^. Notably, the presence of *O*-glycans on V1 were found to reduce virus recognition and neutralization by bNAbs against the V3-glycan epitopes^18^, indicating a role for *O*-glycans, similar to *N*-glycans, in shielding antibody epitopes. Nonetheless, the functions of *O*-glycans on Env remain unclear.

Many studies have shown that *N*-linked glycans on HIV-1 Env interact with glycan-binding proteins (lectins) to impact virus infection and transmission. Algae or plant lectins specific for high mannose-type *N*-glycans, including griffithsin (GRFT), scytovirin (SVN), cyanovirin-N (CVN), and *Galanthus nivalis* agglutinin (GNA), have varying levels of inhibitory activity against HIV-1 isolates^22^. On the other hand, innate immune lectins on host cells that recognize the same or similar *N*-glycan structures can promote HIV-1 infection. For example, DC-SIGN binds high mannose *N*-glycans and is expressed on the surface of certain types of dendritic cells and macrophages at mucosal sites. DC-SIGN can function as a receptor to capture HIV-1 virions and mediate in trans-infection by presenting captured virions to permissive CD4 T cells^23 24, 25^. Siglec-1 is expressed on more mature dendritic cells and also participates in HIV-1 trans-infection through recognition of α2,3 sialic acid on HIV-1^26^. Siglec-7, a cellular lectin with specificity for terminal α(2,8) or α(2,6) sialic acids^27^, binds HIV-1 gp120, and its soluble form has been found to facilitate HIV-1 entry to CD4 + T-cells and macrophages^28^. The mechanism by which soluble Siglec-7 affects virus entry is yet unknown, but it does not facilitate virus adherence to target cells as the membrane-bound Siglec-7 or Siglec-1 do^28–30^. Lectins are also expressed by different bacteria species at the tips of their fimbriae for adherence to host cell-surface glycans and colonization^31^; however, the interactions between such bacterial lectins and HIV-1 glycans and their consequences on HIV-1 infectivity have not been explored.

In this study, we investigated the interaction between HIV-1 and a panel of bacterial lectins and evaluated their effects on virus infectivity and transmissibility. These lectins are specific for high-mannose *N*-glycans or sialyated *O*-glycans, which were among the glycan types enriched in HIV-1 virions. Moreover, these lectins are expressed at the tips of fimbriae that protrude from the bacterial cell wall, shroud the bacteria surface, and can also be shed into the milieu. In particular we tested lectins from bacteria that are part of the human mucosal microbiota: FimH, a lectin of uropathogenic *Escherichia coli* with high affinity for *N*-linked high mannose Man_5_^32, 33^; Msl from vaginal non-pathogenic *Lactiplantibacillus plantarum* CMPG5300 that binds high-mannose Man_5 – 9_ *N*-glycans^34^; SLBR lectins (SLBR-B, SLBR-H, and SLBR-N) from oral nonpathogenic *Streptococcus gordonii strains* that bind sialylated *O*-glycans^35^. This study revealed the ability of these bacterial lectins to bind HIV-1 Env and elicit different effects on virus infectivity. Notably, the *O*-glycan-specific lectin SLBR-N was found to enhance HIV-1 infection in the context of both cell-free virus infection and a CD4-dependent cell-to-cell viral transfer assay. This enhancement was recapitulated to varying extents by other lectins recognizing *O*-glycans, signifying the critical involvement of *O*-glycan engagement. This study also provided initial evidence for the hypothesis that lectin-mediated engagement of *O*-glycans on HIV-1 Env facilitates HIV-1 infection and transmission by promoting HIV-1 Env binding to the CD4 receptor. Hence, this is the first study to demonstrate a direct interaction between HIV-1 and bacteria through fimbrial lectins and to highlight an important novel role for *O*-glycans in influencing HIV-1 infectivity and transmissibility.

## Results

### Enrichment of high-mannose N-glycans and core 1 and core 3 O-glycans in HIV-1 virions as detected by lectin microarray analysis.

**Figure 1:**
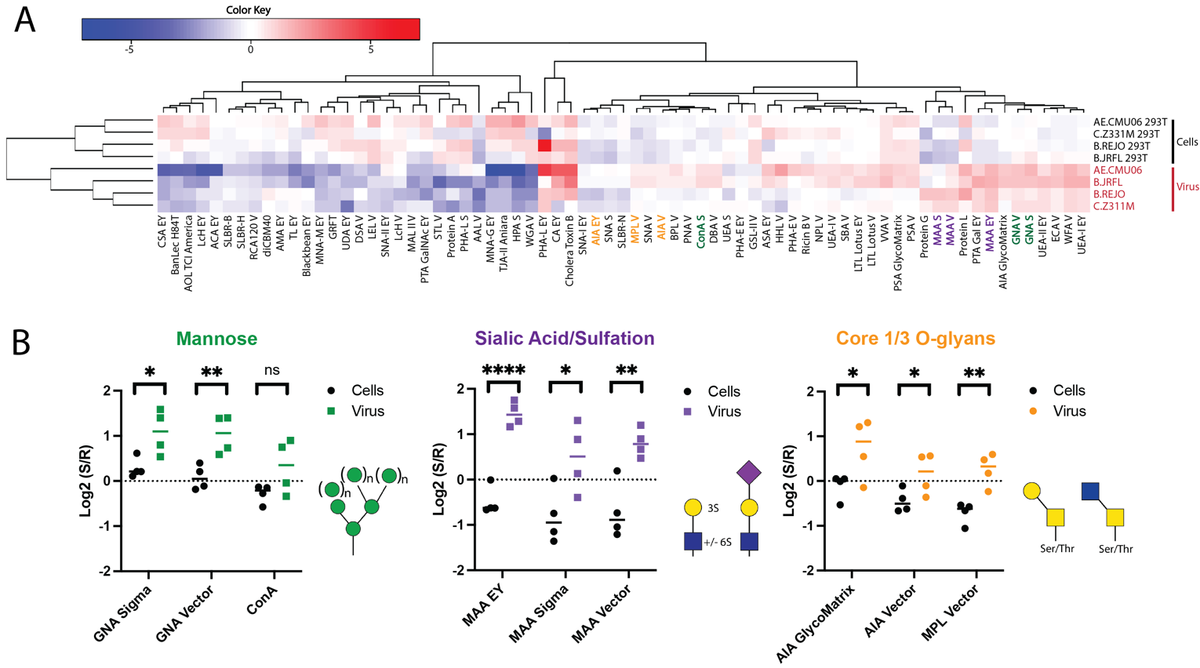
Lectin microarray analysis of HIV-1 lysates versus cell lysates. HIV-1 virions were enriched from supernatants of IMC-transfected 293T cells by centrifugation on sucrose cushion and lysed with 1% Triton X-100 in PBS. The transfected 293T cells were also lysed and tested in parallel. Equal amounts of protein from virus or cell lysates were combined with a pooled reference (R) of all samples and analyzed on the lectin microarray (see **Table S1** for printlist). (A) Heat map of total lectin microarray data for cell and viral lysates. Median normalized log_2_ ratios of (Sample(S)/Reference(R)) are depicted where log_2_(S) > log_2_(R) is shown in red and log_2_(S) < log_2_(R) in blue. Only lectins passing quality control are shown. (B) Mean fold changes for mannose-, sialic acid/suflation-, and O-glycan-specific lectin binding to virus over cell lysates. Three lectins in each category are shown. *, <0.05; **, <0.01, ***, <0.0001.

To understand the biological importance of glycans present on HIV-1 virions, we first determined their glycomic signature using a dual-color lectin microarray technology^36, 37^. We analyzed four virus strains of different clades that included an infectious molecular clone (IMC) with a subtype B chronic envelope (Env) glycoprotein (B.JRFL) or IMCs of acute or transmitted/founder isolates from subtypes B, C and CRF_01.AE (B.REJO, C.Z311M, AE.CMU06). Viral lysates were fluorescently labeled (Alexa Fluor 555), mixed with a pooled reference (Alexa Fluor 647), and applied to the lectin microarray. Viral lysates were tested in parallel with cell lysates of 293T cells from which the viruses were produced in order to detect the relative glycomic variations in virus versus cell samples. Each microarray displayed an identical set of lectins with various glycan specificities immobilized on a glass slide (**Table S1**). By comparing mean fold changes observed in all viral lysates over all cell lysates, we determined the specific glycans that were enriched in virions and deciphered the glycan signatures common to the four HIV-1 strains tested using well curated annotations of lectin specificities^35, 38^. The data in Fig. 1A revealed 35 lectins showing higher binding to virus over cell lysates, and of these, three sets of lectins with distinct glycan specificities are highlighted herein.

The first set is composed of lectins specific for mannose terminated *N*-glycans. Consistent with well documented findings from our lab and others that HIV-1 Env is decorated with high mannose glycans^22,23, 39–41^, we observed increased binding for three mannose-specific lectins (GNA Sigma, GNA Vector, and Con A) to virus versus cell lysates (Fig. 1A) and their statistically significant differences are evident in Fig. 1B (**left panel**). Secondly, we observed a greater binding of MAA lectins to virus lysates which recognize α 2,3 sialic acid or sulfation present on either *N*- or *O*-glycans (Fig. 1A and 1B **middle panel**). The results were observed with MAA lectins from three different sources. The lectin microarray data revealed a third set of lectins with higher binding to virus lysates: AIA GlycoMatrix, AIA Vector, and MPL Vector lectins. All of these lectins are specific for core 1 and core 3 *O*-glycans (Fig. 1A **and** Fig. 1B **right panel**). Multiple *O*-glycosylation sites were predicted to be present in each of the HIV-1 Env strains examined in this and subsequent experiments: most were located in the V1 loop, one in a conserved C5 site upstream from the furin gp120-gp41 cleavage site, and few in the V5 loop and the extracellular gp41 region (**Supplemental Fig. 1**). These data are in line with past studies that experimentally identified *O*-glycans on various Envs^18, 21^ and with previously obtained lectin microarray analysis of HIV-1 virions^37^. Altogether, the data support the notion for an accumulation of high mannose *N*-glycans and core 1 and/or core 3 *O*-glycans on HIV-1 virions relative to the cells producing these viruses.

### Enhanced HIV-1 infectivity upon treatment with lectins binding O-glycans but not with lectins specific for high mannose N-glycans.

We next examined the effects of lectins binding to high-mannose *N*-glycans or *O*-glycans on HIV-1 infection. We focused on lectins expressed by bacteria species that are part of the human mucosal microbiota: high-mannose N-glycan-binding FimH from uropathogenic *E. coli*, high-mannose N-glycan-specific Msl from vaginal *L. plantarum*, and *O*-glycan-specific SLBR lectins (SLBR-B, SLBR-H, and SLBR-N) from oral commensal *Streptococcus gordonii* strains. The binding specificities of these lectins are shown in Table 1. High mannose-specific lectin GRFT, known to have potent anti-HIV-1 activity^22, 23, 42^, was included as a control. *O*-glycan-binding plant lectins (AIA, MAL II) were also examined for comparison. Titrated amounts of each lectin were pre-incubated for 1 hour at 37°C with HIV-1 (AE.CMU06, C.Z311M, or B.REJO). The mixtures were then added to TZM.bl reporter cells and virus infection was measured after 48 hours.

**Figure 2:**
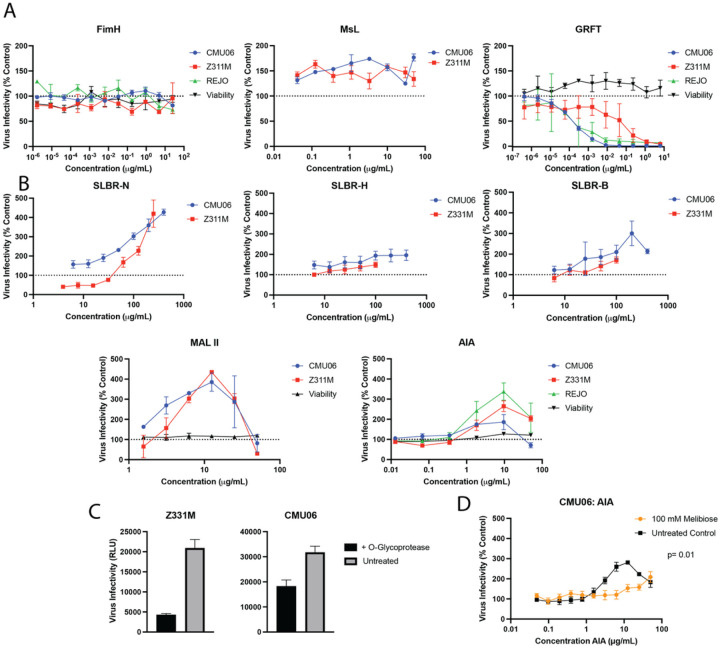
Differential impact of bacterial and plant lectins on HIV-1 infectivity. (A-B) HIV-1 virions were pre-incubated with titrated amounts of lectins for 1 hour at 37° C and assessed for infectivity in TZM.bl reporter cells after 48 hours. Untreated virions served as control (100% infectivity). Full length infectious molecular clones of HIV-1 tested included AE.CMU06, B.JRFL, B.REJO, and C.Z331M. Three lectins specific for high-mannose (A) and five lectins specific for *O*-linked a2,3 sialic acid or core 1 or core 3 *O*-glycans (B) were tested. Cell viability was also measured using PrestoBlue HS Cell Viability reagent (Invitrogen). (C-D) To validate the presence of *O*-glycans and verify that the enhancing effect of *O*-glycan-specific lectins was glycan dependent, virus was pretreated with O-glycoprotease (C) or AIA was pretreated with a saturating concentration of melibiose (D). Reduced infectivity upon treatment with either O-glycoprotease or melibiose indicated speficic contributions of *O*-glycans. A two-way ANOVA was used to compare treated vs untreated. Error is calculated as SD for 2 or 3 biological triplicates.

The data demonstrate that treatment with FimH or Msl neither inhibited nor enhanced viral infection in a dose-dependent manner, whereas GRFT inhibited all three tested viruses with different potencies, as expected (Fig. 2A). Surprisingly, virus treatment with SLBR-N enhanced virus infection up to 4-fold in a dose-dependent manner. The other two SLBR lectins had only modest effects, increasing infectivity by less than 3-fold. A similar pattern was observed upon virus treatment with two other *O*-glycan-specific plant lectins (MAL II and AIA) although the enhancing effects declined at higher concentrations suggesting a distinct mechanism. Enhanced infection by *O*-glycan specific lectins (SLBR-N, AIA, and MAL II) was maintained whether the viruses were produced in 293T cells or PBMCs (**Supplemental Fig. 2**). The presence of GST tag also had no significant effect on enhancement of infection (**Supplement** Fig. 3). The presence of *O*-glycans on HIV-1 was further verified by reduction of virus infectivity upon treatment with O-glycoprotease, an enzyme that cleaves the peptide bond N-terminal to a Ser or Thr containing an *O*-glycan (Fig. 2C). To determine the specific contribution of lectin-glycan interactions to the observed increase of virus infectivity, we pretreated virus with melibiose, a disaccharide that binds AIA^43^. Treatment with melibiose tempered the enhancement of virus infectivity (Fig. 2D). These data suggest that the engagement of *O*-glycans on HIV-1 virions by different lectins including bacterial SLBR-N from oral-colonizing *Streptococcus gordinii* resulted in increased virus infectivity, while the interaction with high-mannose N-glycan-binding bacterial lectins FimH and Msl did not affect infectivity.

### Capture of HIV-1 virions by bacterial lectins specific for O-glycans and high-mannose N-glycans.

**Figure 3:**
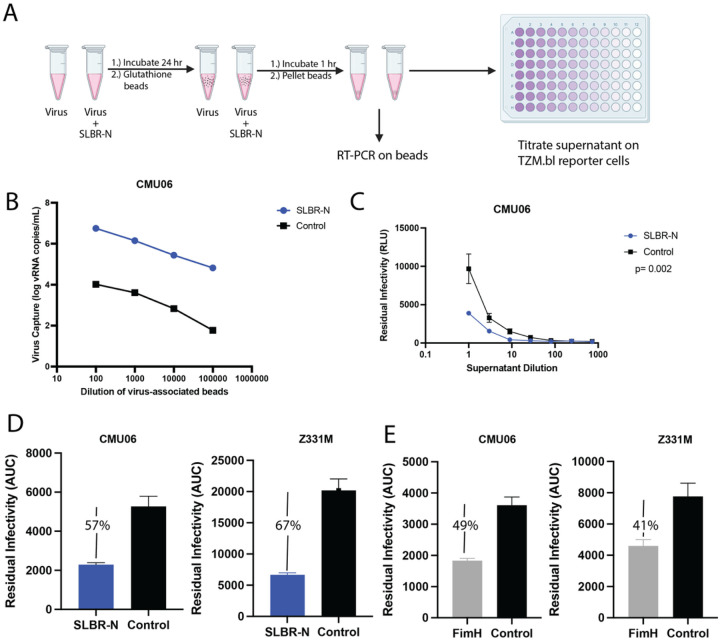
Capture of HIV-1 virions by the SLBR-N lectin. (A) Schematic of the experimental procedure. Virus was incubated with GST-tagged SLBR-N for 24 hours at 37° C and then glutathione beads were added. After the beads were pelleted, RT-PCR was performed on the beads while the supernatant was titrated on TZM.bl cells to measure residual infectivity. (B) The amount of CMU06 virus captured by SLBR-N-coated beads or control beads as measured by qRT-PCR. (C) The residual infectivity of CMU06 virus in the supernatant after capture with SLRB-N-coated beads versus control. A two way ANOVA was used to compare SLBR-N vs control. Areas under the titration curve were calculated and presented in panels D-E. (D-E) The residual infectivity of CMU06 (CRF_01.AE) and Z331M (clade C) in the supernatants after capture with SLRB-N-coated (D) or FimH-coated (E) beads versus control beads. The percentages of HIV-1 captured by each lectin vs control are shown above the bar graph for each lectin.

We next tested whether bacterial lectins can engage HIV-1 virions via viral surface glycans using a virus capture assay outlined in Fig. 3A. Virus was incubated with GST-tagged SLBR-N for 24 hours at 37°C. The mixture was then incubated with glutathione beads and the virion bound beads were pelleted. The beads were washed to remove unbound virions and subjected to qRT-PCR, while the supernatant was titrated on TZM.bl cells to measure residual virus infectivity. The same of input virus was treated with beads in the absence of SLBR-N and analyzed in parallel to serve as a control.

We detected higher levels of viral RNA in beads with SLBR-N-treated virus versus control as measured by qRT-PCR (Fig. 3B), indicating the capture of HIV-1 particles by SLBR-N. Conversely, residual infectivity of the corresponding supernatant was reduced compared to control (Fig. 3C), providing supporting evidence for SLBR-N interaction with *O*-glycans on the virion surface. These experiments were performed with two transmitted/founder isolates (AE.CMU06 and C.Z331M) and comparable results were observed (Fig. 3D). When the relative levels of virus capture were calculated based on the areas under the titration curve of residual infectivity in the supernatant, 57% and 67% capture was observed for CMU06 and Z331M, respectively, upon SLBR-N treatment. We also examined FimH in this assay and found FimH treatment reduced infectivity by 49% for CMU06 and 41% for Z311M (Fig. 3E). Taken together, the data show that both bacterial lectins–α2–3 sialylated *O*-glycan-specific SLBR-N and high mannose *N*-glycan-specific FimH–interacted with the surface of HIV-1 virions, but they had differential effects on virus infectivity.

### Glycan-dependent recognition of HIV-1 Env by bacterial lectins SLBR-N and FimH.

**Figure 4:**
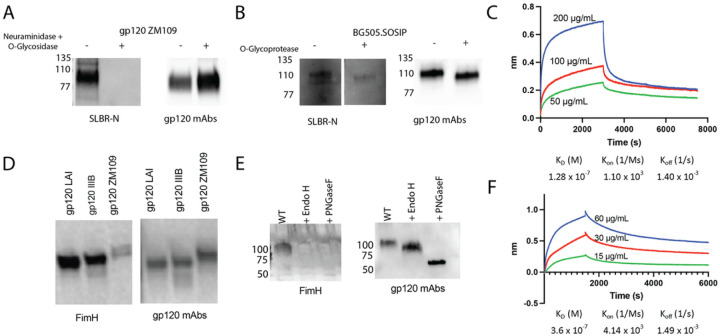
Binding of bacterial lectins to HIV-1 Env in a glycan-dependent manner. (A-B) Recombinant ZM109 gp120 (A) or BG505 SOSIP (B) Env proteins were subjected to SDS-PAGE, transferred to a nitrocellulose membrane and probed with SLBR-N lectin or an anti-gp120 mAb pool. The Env proteins treated with a mixture of neuraminidase and O-glycosidase to remove sialic acids and *O*-glycans or with an O-glycoprotease were tested in parallel. Loss of SLBR-N reactivity verified the *O*-glycan-dependent interaction between SLBR-N and HIV-1 Env. (C) The binding affinity of SLBR-N for HIV-1 gp120 ZM109 as determined using Octet’s BLI. Anti-gp120 mAb 2219 was applied to anti-hIgG Fc biosensors followed by addition of gp120 ZM109. SLBR-N was then reacted at the designated concentrations. (D) Recombinant gp120 proteins (LAI, IIIB, and ZM109) were subjected to lectin blotting as described above and probed with FimH or an anti-gp120 mAb pool. (E) Recombinant gp120 ZM109 pre-treated with EndoH or PNGaseF to remove high mannose *N*-glycans or total *N*-glycans, respectively, was probed with FimH and an anti-gp120 mAb pool. Loss of FimH reactivity indicated that FimH binding to gp120 depended specifically on high mannose moieties on *N*-glycans. (F) The binding affinity of FimH for HIV-1 gp120 ZM109 as measured using Octet’s BLI. Anti-gp120 mAb 2219 was applied to anti-hIgG Fc biosensors followed by addition of gp120 ZM109 and FimH at the designated concentrations.

To determine whether the bacterial lectins SLBR-N and FimH might interact with HIV-1 via the virus Env glycoprotein, we performed a lectin blot assay using recombinant gp120 or SOSIP proteins. SLBR-N was used to probe gp120 ZM109 (Fig. 4A) and BG505 SOSIP (Fig. 4B), which were left untreated or pretreated with a mixture of neuraminidase and O-glycosidase to remove sialic acids and O-glycans or with O-glycoprotease (Fig. 4A, B). The blots were subsequently stripped and re-probed with an anti-gp120 mAb pool to verify the Env bands. SLBR-N recognized untreated gp120 and SOSIP Env proteins, and its reactivity was depleted with enzyme treatment, indicating the interaction of SLBR-N with HIV-1 Env that depends on the presence of *O*-glycans. Biolayer interferometry (BLI) also showed the concentration-dependent kinetics of SLBR-N binding to gp120 ZM109 with a K_D_ of 128 nM (Fig. 4C). These data indicate that SLBR-N recognizes α2–3 sialylated O-glycans on HIV-1 Env.

For comparison, we also subjected recombinant gp120 proteins (LAI, IIIB, ZM109) to lectin blot and BLI analyses with FimH. FimH recognized different gp120 proteins to varying degrees (Fig. 4D), consistent with past data showing the heterogenous presence of high-mannose N-glycans on HIV-1 gp120 proteins. Treatment of gp120 ZM109 with EndoH or PNGaseF enzymes that removed high mannose or all *N*-glycans, respectively, abrogated FimH recognition (Fig. 4E), indicating that high mannose N-glycans on Env are required for FimH-Env interaction. The blots were re-probed with an anti-gp120 mAb pool to define the Env bands and verify the enzymatic glycan cleavage. FimH was also serially titrated and analyzed for its binding affinity to gp120 ZM109 by BLI. We measured a K_D_ of 360 nM, in line with a past report of FimH affinity for Man_7_ glycans^33^. These data altogether demonstrate that both SLBR-N and FimH interacted with HIV-1 virions and the virus Env in particular, but they had distinct impacts on virus infectivity. SLBR-N binding resulted in enhanced virus infection, whereas upon FimH binding virus infectivity was unchanged.

### Enhanced virus infection upon SLBR-N treatment of HIV-1 virions and not target cells.

**Figure 5:**
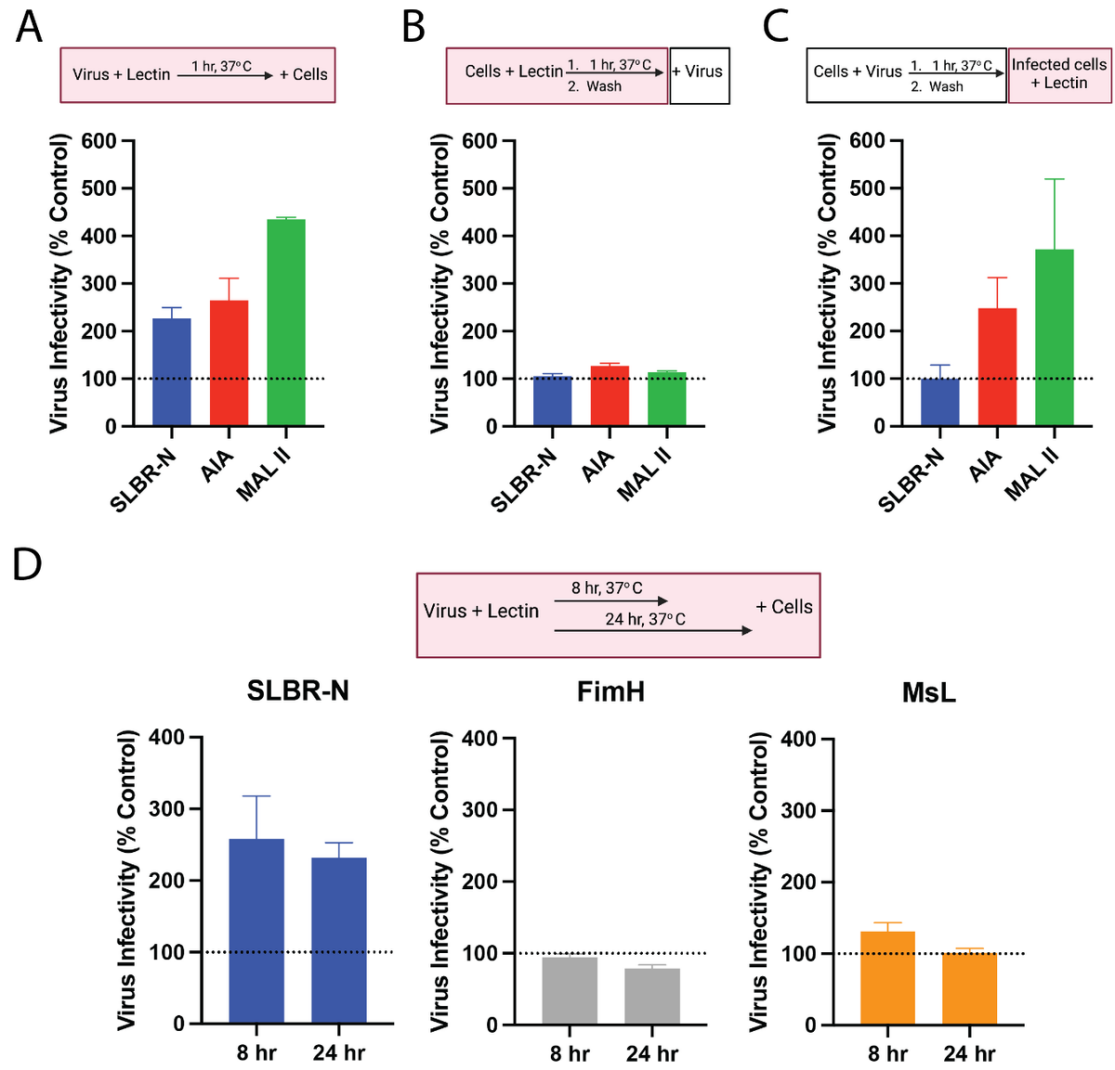
Incubation of virus and bacterial lectin prior to infection is required for enhancement effect. (A) Enhanced infection was observed when HIV-1 Z331M was pretreated with SLBR-N (100 μg/mL), AIA (12.5 μg/mL) and MAL II (12.5 μg/mL) for 1 hour at 37° C and then added to TZM.bl cells. (B) No enhancement was observed when TZM.bl cells were pretreated with SLBR-N, AIA, and MAL II at the aforementioned concentrations for 1 hour at 37°C, washed, and then infected with HIV-1 Z311M. (C) Enhanced infection was observed with AIA and MAL II but not SLBR-N, when the lectins were added at the above concentrations to TZM.bl cells already infected with HIV-1 Z311M. (D) Enhanced infection was stably detected upon prolonged pretreatment of HIV-1 Z311M with SLBR-N (100 μg/mL) for 8 or 24 hours at 37°C, but not with FimH (25 μg/mL) and MsL (25 μg/mL). For all the above experiments, the relative levels of virus infection in the TZM.bl cells were measured at 48 hours by luminescence intensity. Virus infectivity was normalized to the untreated control in each experiment (set to 100%). Experiments were performed in duplicate. Boxes above the graphs show the experimental outlines with red boxes highlighting the presence of lectin in the experiments. Error is calculated as SD for duplicate experiments.

To better understand the mechanism by which the *O*-glycan-binding lectins SLBR-N, AIA, and MAL II augment HIV-1 infectivity, lectin treatment was applied at different time points during the infection assay as depicted in Fig. 5. When lectin was added to the virus first and then mixed with the TZM.bl target cells, an enhancement of virus infection was observed with all three lectins as compared to the untreated control (Fig. 5A). In contrast, treatment of target cells with each of the three lectins prior to infection resulted in no enhancement (Fig. 5B), confirming the importance of lectin interactions with HIV-1 rather than target cells in promoting virus infectivity. Interestingly, when the cells were infected with virus first and then treated with lectins, enhanced infection was observed upon treatment with the two plant lectins AIA and MAL II, but not with SLBR-N, indicating that different steps of virus infection are affected by the plant versus bacterial lectins, which have different numbers of carbohydrate-binding sites and target distinct *O*-glycan structures (Table 1). Taken together this time-course experiment shows that, unlike AIA and MAL II, the bacterial lectin SLBN-N enhanced HIV-1 infectivity mainly by interacting with the virus prior to infection of target cells.

Subsequent experiments were performed to further investigate the impact that prolonged virion-lectin interactions have on virion infectivity. To this end, time course experiments were performed where HIV-1 virions were subjected to a preincubation with bacterial lectins SLBR-N, FimH, or Msl for 8 or 24 hours at 37°C prior to addition of TZM.bl target cells. SLBR-N pretreatment for both 8 and 24 hours led to enhanced virus infection as compared to untreated control (Fig. 5D), recapitulating the enhancement seen with 1 hour pretreatment (Fig. 5A). However, high mannose-specific lectins FimH and Msl had no impact even with prolonged preincubation. These data suggest the specific contribution of *O*-glycan-specific bacterial lectin SLBR-N in promoting the stability and infectivity of HIV-1 virions.

### Enhanced transfer of HIV-1 virions from cell to cell by SLBR-N.

**Figure 6:**
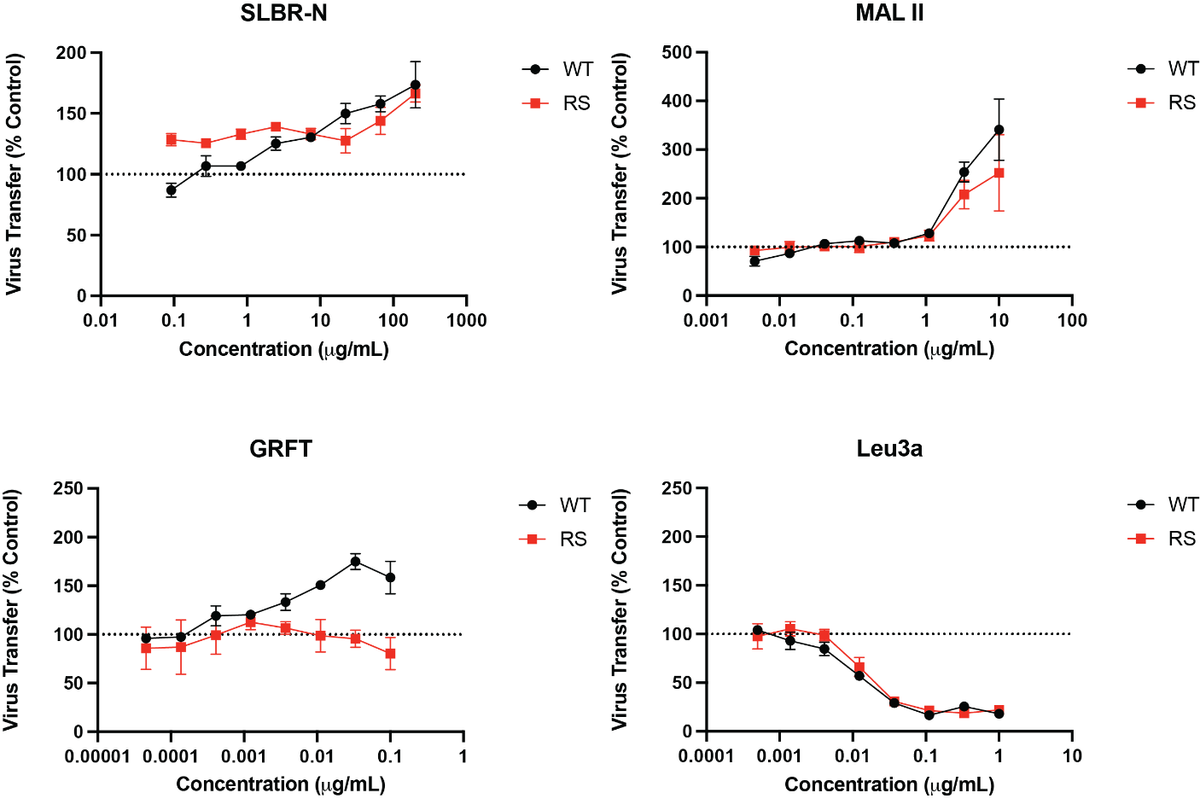
*O*-glycan-specific lectins enhance cell-cell transfer of HIV-1 at the Env-CD4 binding step. Jurkat cells nucleofacted with an HIV-1 clone bearing T/F Env B.QH0692 and an mCherry reporter gene were used as donor cells, whereas primary CD4 T cells served as target cells. The two cell types were mixed for 3 hours, and mCherry+ HIV-1 transfer to target cells was monitored by flow cytometry. Donor cells were pre-treated with titrating amounts of lectin before mixing with target cells. *O*-glycan-specific lectins SLBR-N and MAL II were tested in comparison with high mannose *N*-glycan-binding lectin GRFT. Anti-CD4 Leu3a mAb blocking gp120-CD4 engagement was tested as control. Viruses with WT Env or cleavage-defective RS Env (R508S/R511S) capable of binding CD4 but not virus fusion were tested in parallel. Error is calculed as SEM for two or three repeat experiments.

We also tested the effect of SLBR-N on cell-associated HIV-1 and the transfer of viral particles between cells, a highly efficient mode of HIV-1 spread in vitro^44, 45^. A 3-hour cell-to-cell virus transfer assay was performed using Jurkat T cells nucleofected with an HIV-1 clone bearing T/F Env B.QH0692 with a Gag-iCherry reporter as a donor cell. This clone produces intact virus particles that are highly fluorescent and allow viral particle transfer to be tracked by flow cytometry. The donor cells, which express HIV-1 Env at the cell surface, are then cocultured with primary CD4 T cells allowing for virological synapse formation upon HIV-1 Env/CD4 recognition^44^. The two cell types were also labelled with distinct dyes (eFluor 450 and 660)^46^ to discriminate target cells from donors. Prior to co-culturing, donor cells were incubated with SLBR-N, MAL II, or GRFT. After 3 hour of co-incubation, mCherry + virion transfer to target cells was monitored by flow cytometry. Treatment of donor cells with SLBR-N increased mCherry + WT virion transfer to CD4 T cells (Fig. 6). Enhancement was similarly seen with *O*-glycan-specific MAL II and high-mannose *N*-glycan-specific GRFT. Virus transfer was blocked by the anti-CD4 mAb Leu3a included as a control, which indicates that the cell-to-cell transfer requires Env-CD4 engagement. We then investigated the role of *O*-glycan mediated enhancement in virus-CD4 binding prior to fusion using a gp120-gp41 cleavage-defective virus (due to R519S/R522S (RS) mutations at the REKR furin cleavage site)^47, 48^. Interestingly, only O-glycan-binding lectins (SLBR-N and MAL II) demonstrated enhancement, while transfer of virus in the presence of GRFT was diminished, suggesting distinct mechanisms. Because the RS mutant is capable of binding CD4 but not virus fusion, the results point to *O*-glycan-mediated enhancement at the step of virus-CD4 interaction and prior to viral membrane fusion.

### Enhanced CD4-gp120 binding association by SLBR-N.

**Figure 7:**
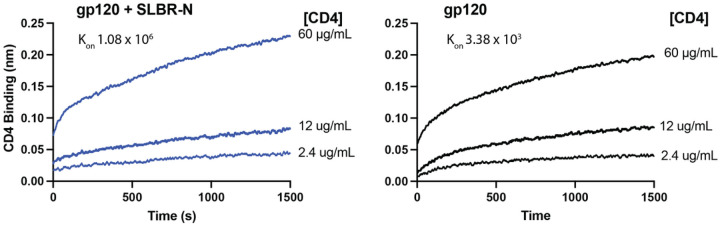
SLBR-N enhances CD4-gp120 binding association as measured by Octet BLI. HIV-1 C.ZM109 gp120 was immobilized on the sensor, treated with SLBR-N (100 μg/mL) or no SLBR-N, and then incubated with soluble CD4 at the designated concentrations. The association rates (k_on_ in M^−1^s^−1^) of CD4 binding to gp120+SLBR-N vs gp120 alone are shown.

Octet BLI was employed to detect changes in CD4 binding to ZM109 gp120 upon treatment with SLBR-N. The data indicate an increase (~ 300-fold) in the binding association (k_on_) of CD4 to SLBR-N-treated gp120 vs. untreated gp120 (Fig. 7). This result suggests that the interactions between SLBR-N and *O*-glycans on gp120 may stabilize the CD4-binding site and promote CD4 binding to gp120.

## Discussion

This study provides evidence for direct interactions between HIV-1 and lectins produced by bacteria present in the host mucosal microbiota, and these interactions impact HIV-1 infection and transmission. Notably, the interactions of HIV-1 with *O*-glycan-binding SLBR lectins that are integral parts of the bacteriafimbriae from commensal oral *Streptococcal gordonii* strains enhanced infectivity of cell-free virions. SLBR-N, one of the SLBRs which recognizes sialyl Lewis X (sLe^X^), displayed the greatest activity. SLBR-N also promoted transmission of cell-associated virus to CD4 T cells. In addition to the SLBR lectins, *O*-glycan-specific plant lectins increased HIV-1 infectivity, indicating a specific effect of *O*-glycan engagement. The mechanisms by which SLBR-N and other *O*-glycan-binding lectins increase HIV-1 infectivity are not fully understood, although our study provides initial evidence that SLBR-N-mediated enhancement may occur at the Env-CD4 binding step as a result of an increased affinity of HIV-1 Env for its CD4 receptor upon lectin-Env interactions. While the in vivo significance of these findings requires more investigation, our study implies a potential role for bacteria that colonize the host mucosa surfaces in influencing HIV-1 infectivity and determining the risk of HIV-1 transmission.

Although core 1 and/or core 3 *O*-glycans have been reported on gp120 from HIV-1, HIV-2, and SIV, the role of HIV-1 *O*-glycans in HIV-1 biology and pathogenesis has been largely understudied. We used a microarray technology with lectins of distinct specificities to identify the enrichment of *O*-glycans and glycans with terminal sialic acids or sulfates as general signatures of HIV-1 virions across different strains. Our experiments with the SLBR lectins further indicated the presence of *O*-glycans with α 2,3-sialic acid and potentially sLe^X^ detected by SLBR-N on the surface of HIV-1 virions and on virus Env glycoproteins. Of note, CD4 + T cells with active HIV-1 replication were found to display higher cell-surface levels of sLe^X^ compared to cells with transcriptionally inactive infection^49^. CD4 T cells with higher sLe^X^ levels also expressed markers associated with HIV-1 susceptibility, as well as intracellular signals known to promote HIV-1 transcription and associate with leukocyte extravasation^49^. It also has been shown that HIV-1 produced in cells deficient of *O*-glycosylation were more sensitive to bNAbs targeting the V3 glycans, indicating the involvement of *O*-glycans in shielding the V3 glycan epitopes. The impact of such epitope shielding on virus escape would be limited, since bNAbs are produced only by a subset of HIV-1 infected individuals^50–52^. Nonetheless, the ability of *O*-glycans to modulate HIV-1 Env recognition and virus neutralization by antibodies more commonly elicited during infection are still unknown. Here we have demonstrated that lectins from bacteria in the human mucosal microbiota can interact with sialyated *O*-glycans on the virus surface and that *O*-glycan recognition specifically resulted in augmented virus infection and cell-cell transfer, signifying a distinctive contribution of *O*-glycans to HIV-1 pathogenesis.

Along with *O*-glycans, high mannose-type *N*-glycans were found to be enriched in HIV-1 virions in line with past reports^39–41^. However, high mannose specific lectins FimH from *E. coli* and Msl from *L. plantarum*, which were studied in tandem with the SLBRs, had little effect on virus infectivity, even though they similarly captured viral particles and recognized HIV-1 Env. On the other hand, high-mannose-specific plant or algae lectins are known to have antiviral activity against HIV-1, including GRFT which is under development for antiviral microbicides^42, 53^. One possible reason for the functional differences between *O*-glycan and *N*-glycan engagement is that, unlike the high density of *N*-glycans on HIV-1 Env, fewer *O*-glycosylation sites are predicted on each Env subunit and they are localized in discrete regions, particularly the V1 loop and at a conserved C5 site near the furin-cleavage site (**Supplemental Fig. 1**), lessening the likelihood for multivalent interactions. Indeed, GRFT is a dimer with multiple putative binding sites per subunit and its antiviral potency has been associated with multivalent interactions with HIV-1 Env^53, 54^, whereas non-inhibitory FimH and Msl lectins are monomers with one glycan recognition site. In the case of *O*-glycan-binding lectins, monomeric SLBR-N and multimeric Jacalin (AIA) and MAL II showed the ability of enhance HIV-1 infection, indicating that multivalency is not requisite for this effect. Rather, we postulate that lectin binding to the particular *O*-glycan location(s) on Env may impact the binding affinity of Env for the CD4 receptor. We also observed that Jacalin and MAL II, but not SLBR-N, enhanced HIV-1 infection upon interacting with cells after virus exposure. In this case, crosslinking of O-glycan-bearing ligands on the cell surface by these multimeric lectins may impart intracellular signal activation to affect cell metabolism and virus replication. Of note, in a report by Silver et al.^18^ Jacalin treatment was shown to inhibit infection of recombinant chimeric NL4–3 viruses, but potent inhibition was seen only for one HIV-1 strain whereas other strains required relatively high lectin concentrations of 250–1000 ug/mL for inhibition, and for one strain enhancement of infection was apparent. In this study, enhancement of infection by Jacalin peaked at 10 ug/mL and was seen with full length IMCs of acute or transmitted/founder HIV-1 strains from different subtypes and with viruses produced in HEK293T or PBMCs. However, we have tested only few IMC strains thus far and Jacalin displayed varying levels of enhancement for the different IMCs, offering the possibility for wide-ranging effects on the highly heterogeneous strains and isolates of HIV-1.

The mechanism driving *O*-glycan-specific lectin engagement to enhance HIV-1 infectivity has not been studied extensively. Our Octet BLI experiments showed that binding of SLBR-N to gp120 enhanced CD4-gp120 binding as indicated by a nearly 2-fold faster on-rate. These results were congruent with the time course and cell-cell viral transfer experiments suggesting that SLBR-N impacts the initial step of virus binding to CD4. The structural explanation for this observation is currently lacking, however a cryo-EM structure of SIV Env trimer, which is highly homologous to HIV-1 Env, in complex with V2-glycan-specific monoclonal antibody PGT145 and Jacalin provides some insight to understand our SLBR-N data. Jacalin was found to attach to each of the three V1 loops in the Env apex (**Supplemental Fig. 4A** and^21^). The V1 loops of most HIV-1 strains we examined are also predicted to have one or more *O*-glycosylation sites (**Supplemental Fig. 1**). Because the V1 *O*-glycan site is in proximity to the CD4-binding pocket in the corresponding Env protomer (**Supplemental Fig. 4B**), lectin engagement to the site may destabilize the Env apex, increasing the accessibility of the CD4-binding pocket. Besides viral Env, *O*-glycan-bearing cell membrane proteins such as CD162 (P-selectin glycoprotein ligand-1), CD43 (sialophorin), and CD44 (E-selectin ligand), are also present on the surfaces of HIV-1 virions^55–57^. The extent to which SLBR-N and similar lectins bind to *O*-glycans on cellular proteins relative to Env and impact virus infection remains unknown and requires further examination.

This study revealed that SLBR lectins from oral Streptococcal bacteria can bind HIV-1 virions and augment virus infectivity. Streptococci including SLBR-expressing *S. gordonii* and closely related *S. mitis* strains are among the most common genera that colonize the human oral cavity, the upper gastrointestinal tract and the genitourinary tracts, and are abundantly present in milk^58–63^. Reminiscent of findings observed for a number of enteric viruses^2–9^ and MMTV^10, 11^, direct interactions between HIV-1 and bacteria or bacterial products in the host microbiota may constitute a critical determinant for HIV-1 acquisition through mucosal routes, including mother to child transmission during perinatal and breastfeeding periods and sexual intercourse activities involving oral and urogenital contacts. The study reported here is also pertinent and timely given that HIV-1 glycans are the main targets for antiviral lectins being explored as candidate microbicides^42, 53^ and for many bNAbs under development for HIV-1 prophylactics^64, 65^. However, the present study is limited to in vitro experiments with recombinant purified lectins, and HIV-1 interactions with SLBR lectins as expressed on bacterial fimbriae and their in vivo consequences have not yet been evaluated. Nonetheless, our findings provide an impetus for investigations into HIV-1 interactions with the host microbiota that are requisite for formulating more effective modalities to prevent HIV-1 infection.

## Methods

### Cell lines, lectins, and viruses

TZM-bl cell line was obtained through the NIH AIDS Research and Reference Reagent Program (ARRRP), contributed by J. Kappes and X. Wu. HEK293T cells were obtained from the American Type Culture Collection (ATCC). The TZM-bl cell line was maintained in Dulbecco’s modified eagle medium (DMEM; Lonza) supplements with 10% heat-inactivated FBS, gentamicin (50 μg/mL), and HEPES (25mM) and the HEK293T cell line was maintained in DMEM containing 10% heat inactivated fetal bovine serum, penicillin/streptomycin (100U/mL), and L-glutamine. Jurkat T cells were obtained from Dr. Arthur Weiss from the NIH HIV Reagent Program (HRP) and maintained in complete RPMI medium (RPMI 1640 medium with 10% FBS, 100U/mL penicillin, 100μg/mL streptomycin, and 2mM glutamine). Primary CD4 + T cells were obtained from isolation of human peripheral blood through the New York Blood Center. Isolation was performed with R&D Systems MagCellect Human CD4 + T cell Isolation Kit (Fisher Scientific). Primary CD4 + T cells were maintained in complete RPMI medium.

The recombinant FimH protein, which contains only the lectin domain, was produced in *E. coli* as described in^66^. SLBRs^67^ and Msl^34^ were similarly produced in *E. coli* transformed with the respective plasmids. All plant lectins were purchased from Vector Labs.

The following reagents were obtained through the NIH AIDS Reagent Program, Division of AIDS, NIAID, NIH: HIV-1 Z331M T/F Infectious Molecular Clone from Dr. Eric Hunter and pREJO.c/2864, ARP-11746, contributed by Dr. John Kappes and Dr. Christina Ochsenbauer. Recombinant IMCs of B.JRFL^68, 69^ and AE.CMU06^41^ were obtained from Dr. Jerome A. Zack (UCLA) and Dr. Chitra Upadhyay (Icahn School of Medicine at Mount Sinai), respectively. These IMCs were generated by transfection of HEK293T using jetPRIME^®^ (Polyplus). Supernatants were filtered (0.45 micron) and pelleted through a 20% sucrose cushion by ultracentrifugation. Viral pellets were resuspended in PBS, tittered on TZM-bl cells, aliquoted, and stored at −80°C until use.

The transmitted/founder (T/F) HIV-1 QH0692 Env (HRP Cat #11227, Drs. David Montefiori and Beatrice Hahn) was cloned into pNL4–3 based HIV-1 Gag-iCherry backbone as previously described^70^. To generate a cleavage-defective Env mutant^71^, we introduced mutations at the primary cleavage site R519S and R522S by PCR amplification using CloneAMP (Takarabio). Mutated PCR fragment was inserted into pNL4–3-QH0692 plasmid with EcoRI-HF and MIuI-HF, with the In-Fusion HD Cloning Kit (Fisher Scientific). HIV-1 Gag-iCherry was digested with EcoRI-HF and XhoI-HF, followed by Gibson assembly (New England BioLabs). The mutants were confirmed by Sanger sequencing across all ampli ed regions.

### Lectin Microarray

Lysates were produced from viruses and the cells from which the viruses by treatment with Triton X-100 (1%) in PBS. After centrifugation, lysates were analyzed for protein concentrations using a DC Assay (BioRad). Each lysate sample (10 μg of protein) was labeled with Alexa Fluor 555-NHS. A reference sample created by pooling equal protein amounts from each lysate was labeled with Alexa Fluor 647-NHS. Printing and hybridization were performed as previously described^36, 72, 73^. The print list for our lectin microarray is shown in Supplemental Table 1. Log_2_ values of the average signals for each lectin are median-normalized over the individual subarray in each channel. Hierarchical clustering using the Pearson Correlation Coefficient, heatmap generation, and data analysis was performed using R (version 1.3.109). Relevant glycan structures were determined using the known specificities for each lectin^38^. Glycan changes were highlighted if they were consistent for all viral versus cell lysates, were observed for lectins that share similar binding motifs and their specificity was unambiguous, and at least one of the lectins was statistically significant (as determined by Student t test).

### Virus Infection Assay

Virus was mixed with titrated amounts of each lectin and incubated at 37°C for 1 hr or up to 24 hrs for some experiments. TZM-bl cells were then added (5,000 cells per well with DEAE dextran at 30 μg/mL) and incubated for 48 hrs. Virus infection was detected using britelite^™^ Plus reagent (PerkinElmer) and luminescence was measured using a BioTek Cytation^™^ 3 luminometer. Virus input was pre-determined to produce RLU values between 100,000 and 200,000. RLUs were normalized to untreated controls (set to 100%). Cells were separately treated with lectin and viability was measured using PrestoBlue Cell Viability Reagent (ThermoFisher). Each condition was tested in triplicate.

For some experiment, virus was pre-treated with O-glycoprotease (20 μl, New England Biolabs) or AIA was pretreated with melibiose (Sigma-Aldrich) for 1 hr at 37°C. Melibiose was maintained at a final concentration of 100 mM throughout the assay.

### Virus Capture

Virus was incubated with SLBR-N (20 μg) or left untreated for 24 hrs at 37°C. Pierce^™^ Glutathione Magnetic Agarose (100 μl, ThermoFisher) was added to the mixture and incubated for 1 hr at 37°C and then pelleted. The supernatant was titrated on TZM-bl cells to measure residual virus infectivity. The beads were washed 3 times with PBS to remove unbound virus and subjected to vRNA quantification by real time PCR using the Abbott m2000 System^69^.

### Lectin Blotting

Recombinant HIV-1 Env were resolved on a 4–20% gradient SDS Page gel (Bio-Rad) and transferred to a nitrocellulose membrane using an iBlot 2 transfer device. The membrane was blocked using either SuperBlock^™^ (PBS) Blocking solution (ThermoFisher) or Blocker^™^ BSA 10% in PBS (ThermoFisher). FimH (2 μg/mL) or SLBR-N (1 μg/mL) was then added in blocking buffer and incubated for 1 hr at room temperature. Blots were washed with PBS-T (PBS with 0.05% Tween-20, pH 7.4, 3x, 5 min each) and THE^™^ His Tag mouse antibody (Genscript, 0.5 μg/mL) for FimH or anti-GST mouse antibody (Abcam, 1 μg/mL) for SLBR-N was added in blocking buffer and incubated for 1 hr at room temperature. Blots were washed again in PBS-T (3x, 5min) and incubated with anti-Mouse antibody HRP (1:1000, KPL Antibodies and Conjugates) for 1 hr at room temperature. After final wash, the blots were developed using the ECL substrate (BioRad) and luminescence was detected using a BioRad ChemiDoc^™^ MP imaging system.

To detect Env bands, blots were stripped using Restore^™^ PLUS Western Blot Stripping Buffer (ThermoFisher) to remove lectins, blocked with 5% milk in PBS-T, and probed with a pool of monoclonal human antibodies against HIV-1 Env. Env bands were detected using an anti-Human Ig HRP antibody (1:1000, KPL Antibodies and Conjugates) and visualized as described above.

For some blots, Env (1–5 μg) was pretreated with O-Glycoprotease (1 μl), α2–3,6,8 neuraminidase/O-glycosidase (2 μl each), EndoH (5 μl), or PNGaseF (1 μl) for at least 1 hr at 37°C using denaturing conditions. All enzymes were purchased from New England Biolabs and the manufacturers protocol was used.

### Biolayer Interferometry

Binding kinetics of bacterial lectins for HIV Env were performed by biolayer interferometry using an Octet Red96 instrument (ForteBio/Sartorius). Anti-Env monoclonal antibody 2219^69, 74^ (5 μg/mL) was immobilized on Octet^®^ AHC Biosensors (Sartorius) followed by recombinant gp120 ZM109 (5 μg/mL). Biosensors were then dipped into titrated amounts of either FimH or SLBR-N. This experiment measured the affinity of each lectin for gp120 glycans in a 1:1 stoichiometry. Samples were diluted in PBS supplemented with BSA (0.1% w/v) and Tween 20 (0.02% v/v). A loaded sensor run with a buffer blank was used as reference to correct for drift. Reference curves were subtracted and the data was analyzed with the Octet Data Analysis software by employing a 1:1 homogenous binding model for a global fit analysis for association and dissociation curves.

For experiments to measure association kinetics for CD4 and SLBR-N-bound gp120, Octet^®^ AHC Biosensors that had been loaded with 2219 (5 μg/mL), recombinant gp120 ZM109 (5 μg/mL), and SLBR-N (100 μg/mL) were dipped into serially titrated amounts of CD4 (1:5 starting at 60 μg/mL). A 2:1 heterogeneous ligand binding model for a global fit analysis was used.

### Cell-to-cell Transfer Assay

Jurkat T cells were nucleofected with HIV-1 Gag-iCherry using Cell Line Nucleofector Kit V (Lonza) and incubated overnight at 37°C. After 16–18 hours, live nucleofected Jurkat (donor) cells were isolated via Ficol density gradient centrifugation. Donor and target primary CD4 + T cells were dye-labeled with Cell Proliferation Dye eFluor 670 and eFluor 450 respectively (Invitrogen). 1×10^5^ donor cells were co-cultured with equal number of target cells in a round bottom 96-well plate. After 3 hours, cells were washed and trypsinized to remove surface-attached virus particles and trypsin activity was neutralized with complete RPMI media. Cells were then washed and fixed with 2% paraformaldehyde (PFA) for 20 minutes at room temperature, then run on Attune Flow Cytometer (ThermoFisher). Positive mCherry signal from transferred HIV Gag-iCherry virus particles from eFluor 450 + cell population represents internalized virus particles transferred from cell to target cell. Neutralization of cell-to-cell transfer assay was performed by preincubating lectins or anti-CD4 antibody, Leu3a, with donor cells for 30 minutes at 37°C prior to cell mixing. Leu3a was also preincubated with acceptor cells.
